# Supplementary material for: Culture of Bovine Aortic Endothelial Cells in Galactose Media Enhances Mitochondrial Plasticity and Changes Redox Sensing, Altering Nrf2 and FOXO3 Levels
Source: Antioxidants (Basel). 2024 Jul 20;13(7):873. doi: 10.3390/antiox13070873 (PMC11274175; doi:10.3390/antiox13070873)
Supplement: Supplementary file 1 [file antioxidants-13-00873-s001.zip › antioxidants-3084618-supplementary.pdf]

Supplementary Material

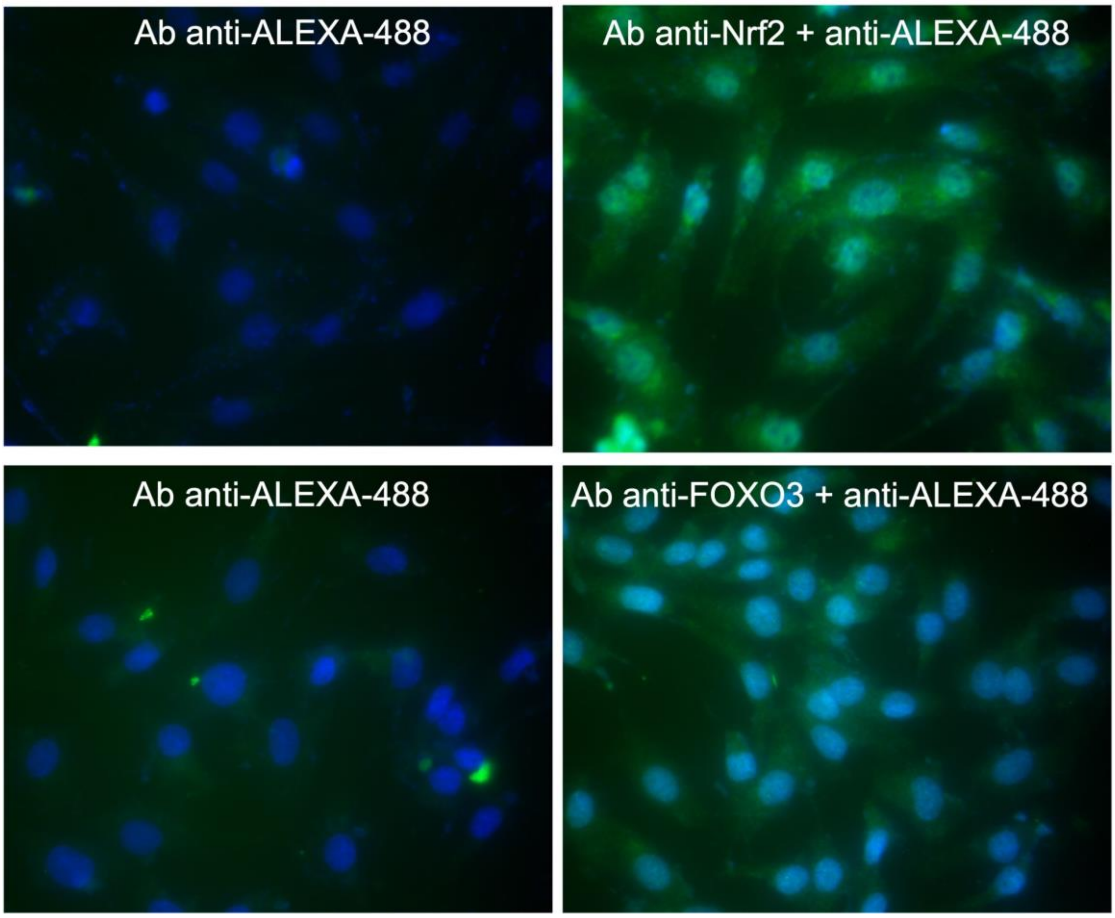

Supplementary Figure S1 Secondary antibody control in BAECS. Representative image of immunofluorescence from Nrf2 and FOXO3 (green) and DAPI (blue) in BAEC after 3 h of incubation with galactose medium. AC1 corresponds to primary antibody and AC2 corresponds to secondary antibody.

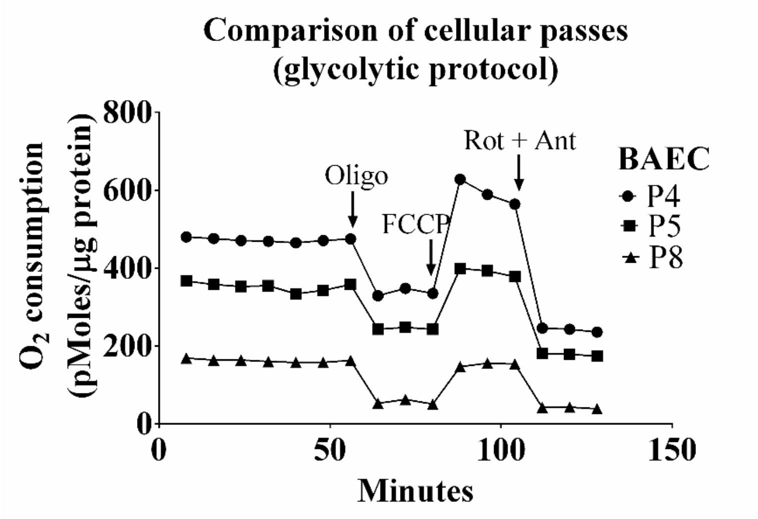

Supplementary Figure S2 Effect of cell passage on mitochondrial respiration in BAEC. Representative respirometry assay of BAEC in different cell passages P4, P5 and P8 in medium containing glucose. Arrows indicate the time of adding the oligomycin, FCCP and rotenone plus antimycin. Plots of O_2 consumption (pMoles) quantification was corrected using total µg of protein.

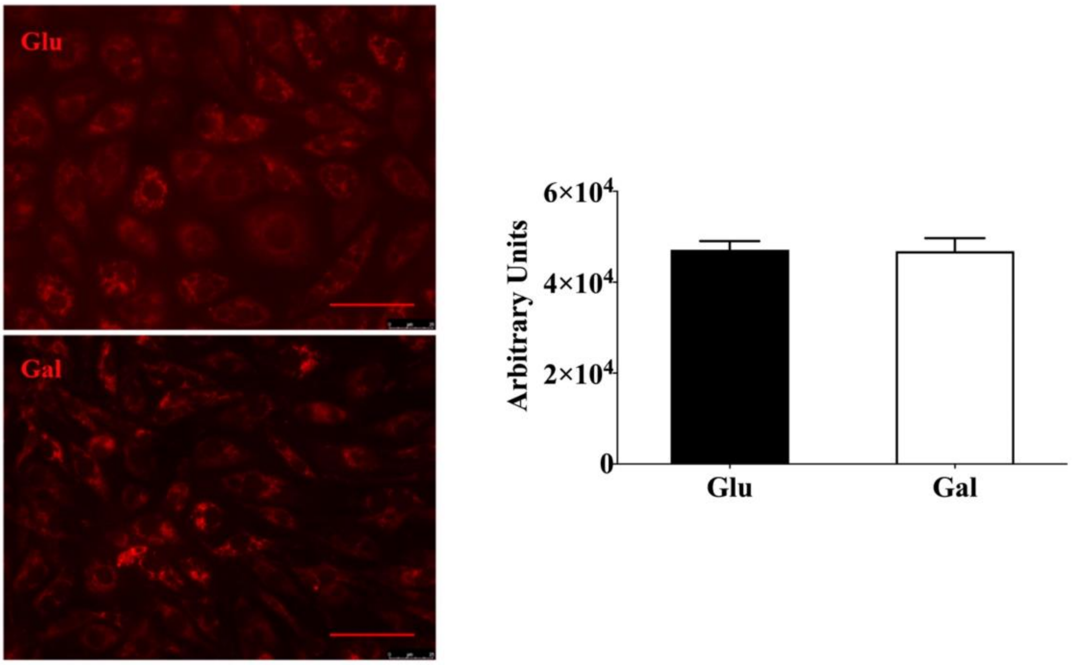

Supplementary Figure S3 Effect of glucose- or galactose-containing medium on mitochondrial O_2^- production in BAEC (P4-6). MitoSOX Red labeling of fixed BAEC by microscopy, showing mitochondrial superoxide levels in BAEC conditioned in media containing glucose or galactose for 24 h. Each independent experiment was performed using cells in the same cell passage exposed to glucose or galactose media. The red bars represent 100 μm. Data in graphs represent mean ± SEM (n=4). * $p < 0.05$, indicate statistical difference between the groups of glucose (Glu) and galactose (Gal) by *t* test.

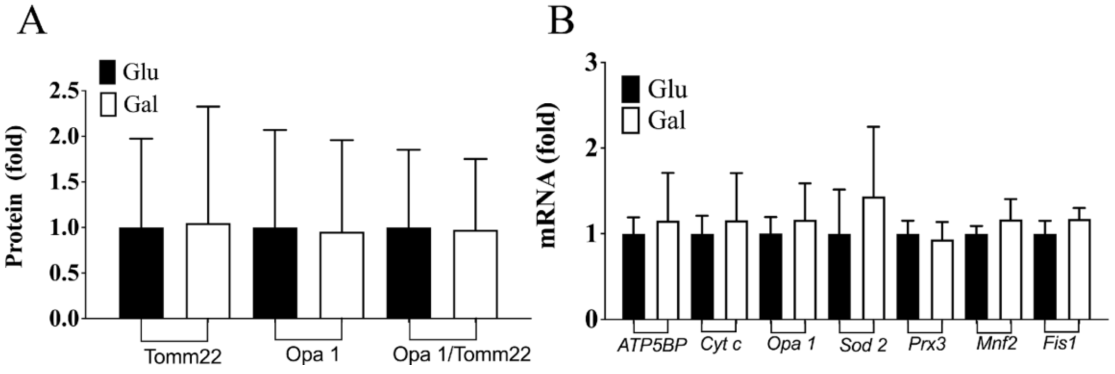

Supplementary Figure S4 Effect of glucose- or galactose-containing medium on mitochondrial content in BAEC (P4-P8) at 24 h. (A) WB analysis of mitochondrial proteins Tomm22 and Opa1 and the ratio Opa1/Tomm22. (B) Gene expression changes in ETC components (*Cyt c*, *ATP5BP*), mitochondrial antioxidants (*Sod2*, *Prx3*) and mitochondrial dynamics regulators (*Fis1*, *Mnf2*, *Opa1*) coding genes. Each independent experiment was performed using cells in the same cell passage exposed to glucose or galactose media. Data in graphs represent mean ± SEM (n=5/WB, n=4/qRT-PCR).

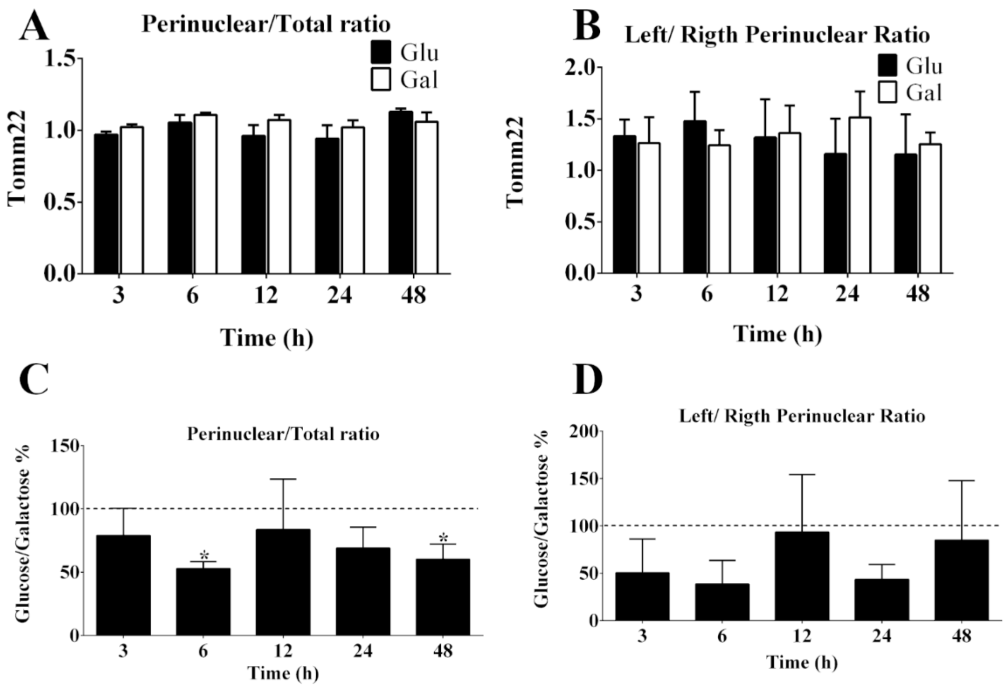

Supplementary Figure S5 Effect of glucose- or galactose-containing medium on mitochondrial dynamics and intercellular variability in BAEC (P4-6). (A) Perinuclear/total ratio, (B) left/right intensity ratio. Intercellular variability was evaluated as the magnitude of standard deviation for: (C) perinuclear/total ratio, (D) left/right intensity ratio. Each independent experiment was performed using cells in the same cell passage exposed to glucose or galactose media. Data in graphs represent mean ± SEM (n=4). * $p < 0.05$, indicate statistical difference between the groups of glucose (Glu or dashed line) and galactose (Gal or black bars) by two-way ANOVA, followed by the Bonferroni's *post hoc* test.

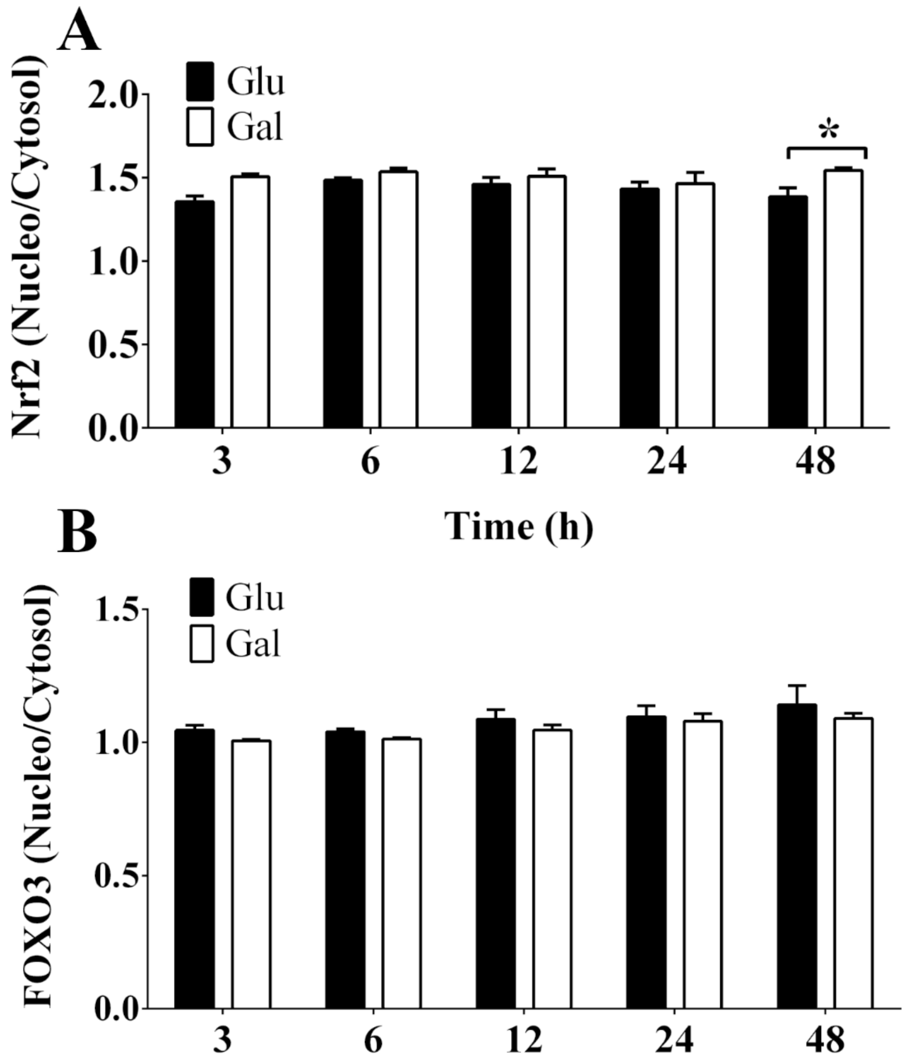

Supplementary Figure S6 Effect of glucose- or galactose-containing medium on Nrf2 or FOXO3 translocation in BAEC (P4-6). (A) Nrf2 nucleus/cytosol ratio, (B) FOXO3 nucleus/cytosol ratio. Each independent experiment was performed using cells in the same cell passage exposed to glucose or galactose media. Data are represented as mean ± SEM (n=4). **p*<0.05 indicate statistical difference between glucose (Glu) and galactose (Gal) conditions, by two-way ANOVA, followed by the Bonferroni's *post hoc* test.
